# DID CHILDHOOD ABUSE PREDICT CARE EXPECTATION FOR FORMAL CARE: THE ROLES OF FAMILY FUNCTIONING AND CARE PREPARATION

**DOI:** 10.1093/geroni/igad104.0137

**Published:** 2023-12-21

**Authors:** Xue Bai, Chang Liu, Jiajia Zhou, Bo Hu

**Affiliations:** The Hong Kong Polytechnic University, Hong Kong, Hong Kong; The Hong Kong Polytechnic University, Hong Kong, Hong Kong; The Hong Kong Polytechnic University, Hong Kong, Hong Kong; London School of Economics and Political Science, London, England, United Kingdom

## Abstract

Objectives: The influence of childhood abuse could be lasting and extensive. This study aims to examine the associations between childhood abusive experiences (i.e., physical abuse, emotional abuse, and bully victimization) and ageing adults’ care expectation for formal care in later life, as well as the potential mechanism of the associations. Methods: This study used data from the Panel Study of Ageing and Society (PAAS) in Hong Kong, with 5,007 participants aged 50 and over included in the study. Path analyses were performed to examine the direct effects of abusive experiences in childhood on future care expectation and formal care, and the indirect effects through serial mediators of family functioning and care preparation. Results: Ageing adults experiencing more childhood abuse were less likely to report care expectations in the future ( = -0.051, p < 0.01), and the direct effects ( = -0.042, p < 0.05) and indirect effects ( = -0.009, p < 0.05) from the serial mediation were significant. Among those people who reported clear care expectations, they were more likely to choose formal care if they had more abusive experiences in childhood (= 0.049, p < 0.05), from which the association was largely explained by decreased family functioning and more care preparation ( = 0.042, p < 0.001). Discussion: This study is the first to connect childhood abuse and late-life care expectation. From a life course perspective, it encourages more considerations regarding care preparation and formal care among people with a history of childhood abuse.

